# Thinking and feeling sustainable: identity-based cognitive and affective mechanisms in food choices across generations

**DOI:** 10.3389/fpsyg.2026.1808114

**Published:** 2026-07-08

**Authors:** Han-Jen Niu, Chun-Ting Lin, Ming-Hsuan Li, Chun-Chieh Yu

**Affiliations:** 1 Department of Management Sciences, Tamkang University, New Taipei City, Taiwan; 2 Department of Industrial and Business Administration, St. John’s University, New Taipei City, Taiwan

**Keywords:** green self-identity, sustainable consumption, sustainable food consumption, purchase intention, cognitive involvement, affective involvement, environmental psychology, generational differences

## Abstract

**Background:**

Sustainable food consumption is often framed as a response to environmental concerns. However, from a psychological perspective, such choices also reflect value-laden decisions shaped by self-identity, cognitive evaluation, and emotional engagement. Despite widespread support for sustainability, a persistent intention–behavior gap remains, suggesting that information alone is insufficient to explain why consumers translate pro-environmental values into actual purchasing intentions. Drawing on identity theory, signaling theory, and social comparison theory, this study proposes a dual-mechanism framework in which green self-identity influences sustainable food purchasing intention through cognitive and affective involvement.

**Methods:**

A survey was conducted among 533 consumers in Taiwan. Structural equation modeling was employed to examine the direct and indirect effects of green self-identity on sustainable food purchasing intention. Mediation analyses were used to assess the roles of cognitive and affective involvement, while multi-group analyses were performed to compare the psychological mechanisms across Generation Y and Generation Z consumers.

**Results:**

The findings indicate that green self-identity positively influences sustainable food purchasing intention both directly and indirectly through cognitive and affective involvement. Cognitive involvement reflects consumers’ analytical evaluation of sustainability-related information, whereas affective involvement captures emotional resonance with sustainability values. The results further reveal meaningful generational differences. Cognitive involvement exerts a stronger influence among Generation Y consumers, whereas affective involvement plays a more prominent role among Generation Z consumers.

**Conclusion:**

This study demonstrates that sustainable food choices are shaped by both thinking and feeling processes through which identity is translated into behavioral intention. By integrating cognitive and affective mechanisms within an identity-based framework, the study advances understanding of sustainable consumption decision-making. The findings also underscore the importance of generational differences, suggesting that sustainability communication strategies should align with the distinct cognitive and emotional orientations of different consumer cohorts.

## 1 Introduction

Sustainable food consumption has become a central issue in contemporary consumer research. It is often framed as a response to environmental concern. At the global level, sustainability has shifted from a technical issue to a broader societal and economic priority, with increasing emphasis on consumption-driven transitions (Andrade and Vieites, 2025; Nguyen et al., 2026). Within this context, sustainable food consumption represents a key consumer-level pathway linking environmental responsibility and market behavior (Rex and Baumann, 2007).

From a psychological perspective, however, such choices are not merely reactive. They are constructed through processes of interpretation, identity alignment, and emotional engagement. Consumers do not simply respond to sustainability information; they interpret it, relate it to who they are, and decide whether to act on it. This shift suggests that sustainable consumption is fundamentally a psychologically mediated process rather than a purely informational outcome.

Prior research has emphasized the role of self-identity in shaping pro-environmental behavior. In particular, green self-identity refers to the extent to which individuals perceive environmental responsibility as part of their self-concept. Individuals with a strong green self-identity are more likely to engage in value-consistent behavior (Becerra et al., 2023; Kumar et al., 2023; Niu and Chen, 2022). However, most studies treat identity as a direct predictor of behavioral intention, leaving unclear how identity is translated into decision-making processes. As a result, the underlying psychological mechanisms remain insufficiently specified.

This limitation is reflected in the well-known intention–behavior gap. Consumers often express strong environmental concern and positive attitudes toward sustainability, yet these intentions do not consistently translate into sustainable food choices (Rex and Baumann, 2007). This gap cannot be explained by information availability alone. Rather, it reflects differences in how consumers evaluate, interpret, and internalize sustainability-related cues. Recent research further suggests that sustainable food consumption is shaped not only by environmental attitudes, but also by the level of consumer involvement and psychological engagement with sustainability-related issues (Niu et al., 2025a).

To address this issue, it is necessary to examine the mechanisms linking identity and behavior. Building on recent advances in sustainable consumption research (Andrade and Vieites, 2025; Nguyen et al., 2026), this study focuses on two complementary pathways: cognitive involvement and affective involvement. Cognitive involvement captures how consumers analytically evaluate the credibility and relevance of sustainability cues, whereas affective involvement reflects how such cues are internalized into emotionally meaningful and identity-consistent experiences. Importantly, these mechanisms represent complementary psychological pathways linking identity to sustainable food purchasing intention. Cognitive evaluation provides a legitimacy basis, while affective involvement sustains behavior through internalization (Cancellieri et al., 2022).

These processes are further shaped by the social environment. Sustainability-related cues are increasingly embedded within socially visible and digitally mediated contexts. Recent research also suggests that sustainability-related behaviors are socially embedded and influenced by relational and community-based participation processes (Niu et al., 2025b). Within such contexts, social connection and interaction shape how individuals interpret and engage with sustainability-oriented practices.

To explain these socially embedded processes, this study integrates Signaling Theory and Social Comparison Theory. Signaling Theory explains how eco-labels function as credibility signals that reduce information asymmetry and guide cognitive evaluation (Abubakari et al., 2025; Spence, 1973). In contrast, Social Comparison Theory explains how individuals interpret and enact sustainability through socially referenced identity processes, thereby strengthening emotional engagement (Festinger, 1954; Jang and Kim, 2024). Together, these perspectives provide a coherent explanation of how sustainability cues activate both cognitive evaluation and affective internalization mechanisms.

Importantly, these mechanisms do not operate uniformly across consumers. Generational differences shape how sustainability information is interpreted and enacted. Generation Y tends to emphasize information credibility and functional evaluation, whereas Generation Z is more likely to engage with sustainability as a form of identity expression and social belonging (Dabija et al., 2019; Becerra et al., 2023). These differences influence the relative salience of cognitive and affective pathways, functioning as theoretically meaningful boundary conditions.

Despite growing research on green self-identity and generational differences, an important gap remains. Prior studies have rarely examined how identity, dual psychological mechanisms, and generational context operate together. Most research considers these elements separately, without specifying their interaction. As a result, it remains unclear how identity-driven processes jointly translate into sustainable food purchasing decisions.

To address this gap, this study develops and tests an identity-based dual-mechanism model linking green self-identity to sustainable food purchasing intention through cognitive and affective involvement, while incorporating generational differences as a moderating condition. Using survey data from Taiwanese consumers and applying structural equation modeling with mediation and multi-group analysis, this study examines how these mechanisms operate and vary across Generation Y and Generation Z.

Against this backdrop, this study makes three contributions. First, it clarifies how cognitive and affective involvement function as sequential and interrelated mechanisms linking identity to behavior, thereby addressing the intention–behavior gap. Second, it identifies generational differences as boundary conditions that shape the relative importance of these mechanisms. Third, it integrates signaling and social comparison perspectives to explain how sustainability cues function as both cognitive signals and identity-relevant meanings in contemporary consumption contexts.

## 2 Literature review

### 2.1 Signaling theory and social comparison theory

Recent research in environmental and consumer psychology suggests that green self-identity is not formed solely through internalized values. Rather, it is continuously shaped through social interaction, observation, and feedback, particularly in digitally mediated environments (Berger and Heath, 2007; Whitmarsh and O’Neill, 2010). From this perspective, sustainable consumption is not merely a functional decision. It reflects an ongoing process of identity construction in socially visible contexts. More broadly, recent work emphasizes that sustainable consumption involves both structural constraints and psychological processes, requiring integrated explanations that connect information processing, trust formation, and identity expression (Andrade and Vieites, 2025; Nguyen et al., 2026).

Signaling Theory explains how sustainability-related attributes gain meaning under conditions of uncertainty. Individuals rely on observable consumption behaviors to convey underlying traits, intentions, or values (Spence, 1973). In this context, eco-labels function as credibility signals that help consumers infer otherwise unobservable product qualities (Grunert et al., 2014). Empirical evidence further shows that trust in eco-labels enhances perceived value and willingness to pay, highlighting the central role of signaling in cognitive evaluation (Abubakari et al., 2025). This cognitive validation process is particularly critical in sustainability contexts, where information asymmetry and credibility concerns are prevalent. This suggests that the influence of sustainability claims is conditional rather than automatic. Growing concerns about greenwashing have increased consumer skepticism, implying that sustainability claims must first be cognitively validated before influencing behavior (Kovač et al., 2025). In this sense, signaling operates as a cognitive filtering mechanism.

Social Comparison Theory complements this perspective by explaining how individuals interpret and regulate behavior through reference to others (Festinger, 1954). In contemporary consumption environments, sustainable behaviors are increasingly visible through peers, influencers, and online communities. Such exposure increases the salience of environmental identity and shapes behavioral expectations. Prior research further suggests that social norms and environmental consciousness significantly influence green purchasing behavior, reinforcing the socially embedded nature of sustainable consumption decisions (Lin and Niu, 2018).

Importantly, this process extends beyond cognitive evaluation. Social comparison also activates affective responses such as pride, belonging, and moral satisfaction, thereby linking external observation to internal meaning (Jang and Kim, 2024; Hamby and Jones, 2022). Through repeated comparison and social feedback, sustainable behaviors become emotionally reinforced and socially legitimized.

Taken together, these perspectives indicate that cognitive and affective involvement are not independent processes. Rather, they function as complementary and mutually reinforcing psychological mechanisms. Only after passing this cognitive threshold can these cues be internalized through social comparison processes, which transform evaluation into emotionally meaningful and identity-consistent experience. Prior research similarly shows that cognitive appraisal can strengthen emotional engagement in food-related decisions (Cancellieri et al., 2022). In this framework, cognition acts as a gatekeeping mechanism, while affect serves as an internalization mechanism.

From this integrated perspective, sustainable food consumption involves two interrelated challenges: information asymmetry and social identity management. On the one hand, consumers must evaluate the credibility of sustainability claims. On the other hand, consumption choices serve as expressions of values and social belonging (Whitmarsh and O’Neill, 2010). Recent studies further suggest that eco-labels are increasingly expected to communicate not only environmental benefits but also broader value propositions, reinforcing their dual cognitive–affective role (Langaro et al., 2025). These dual challenges cannot be explained by a single theoretical lens.

Within this framework, Signaling Theory primarily underpins the cognitive pathway, as eco-labels reduce uncertainty and activate analytical evaluation. In contrast, Social Comparison Theory underpins the affective pathway, as social visibility enhances emotional resonance and identity alignment. Importantly, these pathways are interdependent. Cognitive validation conditions the extent of affective engagement, while affective involvement reinforces and stabilizes cognitively informed decisions.

This mechanism also helps explain generational variation. Generation Y tends to emphasize information credibility and functional evaluation, whereas Generation Z is more likely to engage with consumption as a form of identity expression in socially mediated environments (Dabija et al., 2019; Becerra et al., 2023). Accordingly, signaling processes are more salient in shaping cognitive involvement among Generation Y, while social comparison processes more strongly amplify affective involvement among Generation Z.

In sum, neither Signaling Theory nor Social Comparison Theory alone is sufficient. Their integration explains both how consumers evaluate sustainability cues and why they act on them. This provides a coherent theoretical foundation for the identity-based dual-mechanism framework proposed in this study.

### 2.2 Green self-identity (GSI)

Green self-identity refers to the extent to which individuals incorporate environmental responsibility into their self-concept. As a core construct in environmental psychology, self-identity reflects relatively stable self-narratives that guide how individuals perceive, evaluate, and respond to the world (McAdams, 1995). From this perspective, behavior is not only driven by external stimuli, but is also guided by the need to maintain consistency with one’s self-definition.

Building on this logic, green self-identity has been widely identified as a key predictor of pro-environmental intention and behavior. Individuals who see themselves as environmentally responsible are more likely to interpret sustainability-related cues as personally relevant and to act in ways that align with this identity (Chen and Chang, 2012; Kumar et al., 2023). In this sense, identity operates not merely as an attitude, but as a central organizing framework that shapes both perception and action.

This identity-based mechanism becomes particularly salient in consumption contexts. When consumers encounter eco-labels or sustainability claims, their responses are filtered through their self-concept. As a result, individuals with stronger green self-identity are more likely to assign greater meaning and importance to sustainability-related information. This process strengthens the link between environmental values and actual decision-making by making sustainability personally meaningful.

Generational research further extends this argument by highlighting differences in how identity is expressed. Younger consumers, particularly those in Generation Z, tend to construct and communicate identity in more socially visible ways. Sustainable consumption, in this context, becomes a means of signaling values and belonging. Empirical evidence shows that this cohort is more willing to pay premiums for eco-friendly products and is more responsive to symbolic aspects of sustainability (Dabija et al., 2019; Becerra et al., 2023). Similar patterns have been observed across sustainability domains, including services and energy-related behavior, where social and affective factors play a central role (Mahasuweerachai and Suttikun, 2022).

At a broader level, prior research consistently emphasizes the role of identity in shaping how individuals interpret sustainability-related information. Environmental identity influences not only what consumers value, but also how they process information and translate values into behavior (Whitmarsh and O’Neill, 2010). This perspective reinforces the idea that identity operates as a central cognitive and motivational mechanism within sustainable consumption.

Taken together, green self-identity can be understood as an internalized self-schema rooted in both personal values and social learning. Its development is shaped by factors such as environmental education, peer influence, and social interaction processes, all of which vary across generational contexts (Whitmarsh and O’Neill, 2010; Berger and Heath, 2007; Kumar et al., 2023). Its consequences include stronger purchase intention, higher willingness to pay, and increased responsiveness to eco-label cues (Kumar et al., 2023; Mehta et al., 2024; Lau et al., 2023).

In the present study, green self-identity is therefore positioned as the foundational construct that activates both cognitive and affective involvement. Through these mechanisms, identity is translated into sustainable food purchasing intention by aligning cognitive evaluation with emotional commitment.

### 2.3 Cognitive involvement (CI)

Cognitive involvement refers to the extent to which individuals invest mental effort in processing information they perceive as personally relevant (Zaichkowsky, 1985). In sustainable food consumption contexts, involvement plays an important psychological role in linking sustainability orientation to consumption behavior, particularly when consumers actively evaluate environmentally relevant information and product attributes (Niu et al., 2025a). Higher levels of involvement are associated with more systematic and analytical processing, as suggested by the Elaboration Likelihood Model (Petty et al., 1983). In this sense, cognitive involvement reflects not only attention, but also the depth and structure of information processing.

In sustainability contexts, this processing becomes critical due to pervasive uncertainty. Consumers are often unable to directly verify environmental claims, making eco-labels and sustainability cues subject to evaluation rather than acceptance. Recent research highlights that trust in green labeling and perceived value are key determinants of consumer responses, indicating that sustainability information must first pass a cognitive validation process before influencing behavior (Abubakari et al., 2025). At the same time, increasing awareness of greenwashing further reinforces consumer skepticism, making analytical scrutiny a necessary step in decision-making (Kovač et al., 2025).

Within this framework, cognitive involvement functions as a gatekeeping mechanism. Individuals actively evaluate the credibility, relevance, and consistency of sustainability-related information before integrating it into their decision processes. This role becomes particularly salient when environmental responsibility is incorporated into self-identity. When sustainability is self-relevant, individuals are more likely to engage in deliberate and reflective evaluation of environmental consequences (Van der Werff et al., 2013).

From a self-regulatory perspective, cognitive involvement also supports identity consistency. When individuals perceive a discrepancy between their behavior and their environmental identity, they may increase cognitive engagement to restore alignment (Hu et al., 2014). In this sense, cognitive involvement is not merely an information-processing mechanism. It is a mechanism through which individuals filter, validate, and align sustainability cues with their self-concept.

Taken together, cognitive involvement represents the first stage of identity-based decision-making. It determines whether sustainability cues are considered credible and meaningful, thereby conditioning subsequent psychological processes (Andrade and Vieites, 2025; Nguyen et al., 2026). This extends signaling beyond information transmission, positioning it as a mechanism that structures how sustainability claims are cognitively evaluated under uncertainty.

*H1*: Green self-identity positively influences cognitive involvement.

### 2.4 Affective involvement (AI)

While cognitive involvement emphasizes evaluation, affective involvement captures the emotional significance individuals attach to sustainability-related objects or behaviors (Skinner and Belmont, 1993). It reflects how sustainability is experienced, rather than how it is assessed.

In sustainability contexts, emotional engagement plays a central role in transforming evaluation into commitment. When sustainability-related information evokes emotions such as pride, moral satisfaction, or belonging, individuals are more likely to develop stronger attachment and motivation toward sustainable behavior (Hamby and Jones, 2022). These emotional responses reinforce behavioral intention by embedding sustainability within personally meaningful experience.

Importantly, affective involvement does not arise independently. Building on prior cognitive validation, affective involvement plays a critical role in sustaining behavior by embedding sustainability within identity-consistent emotional meaning. Only when sustainability cues are perceived as credible and relevant can they be internalized into emotionally meaningful constructs. This process is consistent with recent research showing that eco-labels can communicate not only environmental benefits but also broader value propositions, thereby enhancing emotional engagement (Langaro et al., 2025).

From this perspective, affective involvement functions as an internalization mechanism. It transforms cognitively validated information into identity-consistent emotional experience. When sustainability aligns with self-identity, emotional engagement becomes stronger and more stable, reinforcing the connection between values and behavior (Jang and Kim, 2024).

This mechanism explains why emotional engagement often sustains behavior beyond rational evaluation. While cognition determines whether sustainability claims are accepted, affect determines whether they are integrated into the self. In this sense, affective involvement stabilizes and strengthens identity-consistent behavior.

*H2*: Green self-identity positively influences affective involvement.

### 2.5 Sustainable food purchasing intention (SFPI)

Sustainable food purchasing intention reflects an individual’s willingness to choose food products associated with environmental and ethical considerations. This intention does not emerge from a single factor. Rather, it results from the interaction between cognitive evaluation, emotional engagement, and self-identity.

Cognitive involvement contributes to intention by reducing uncertainty and establishing trust. Through analytical evaluation, consumers assess the credibility and value of sustainability claims, forming a rational basis for decision-making (Hou and Sarigöllü, 2022). This process is particularly important in contexts characterized by information asymmetry and skepticism toward environmental claims (Kovač et al., 2025).

However, cognitive evaluation alone is often insufficient. Affective involvement strengthens behavioral intention by embedding sustainability within emotional and identity-relevant meaning. Emotional attachment, moral resonance, and value alignment increase the motivational force behind sustainable choices (Zheng et al., 2023). In this sense, affect transforms evaluation into commitment.

Green self-identity integrates these mechanisms. Individuals who perceive environmental responsibility as part of their self-concept are more likely to act in identity-consistent ways, both directly and through increased involvement (Lau et al., 2023; Mehta et al., 2024). This identity-based process reflects a broader shift in sustainable consumption research, which emphasizes the interaction between structural conditions, cognitive evaluation, and psychological internalization (Andrade and Vieites, 2025; Nguyen et al., 2026).

Importantly, cognitive and affective involvement operate as complementary yet interrelated mechanisms. Cognitive involvement provides evaluative validation of sustainability cues, whereas affective involvement embeds these cues within identity-consistent emotional meaning. Together, these mechanisms explain how consumers translate sustainability-related evaluation into stronger commitment toward sustainable behavior. It also aligns with evidence showing that cognitive appraisal enhances emotional responses in food-related decisions (Cancellieri et al., 2022).

Taken together, sustainable food purchasing intention can be understood as the outcome of a two-stage mechanism:

(1) cognitive validation of sustainability cues, and
(2) affective internalization into identity-consistent motivation.

This integrated process provides a coherent explanation of how identity is translated into behavior. This mechanism provides a theoretical explanation for the intention–behavior gap, demonstrating that behavioral commitment emerges only when cognitively validated information is internalized into identity-relevant emotional meaning.

*H3*: Cognitive involvement positively influences sustainable food purchasing intention.

*H4*: Affective involvement positively influences sustainable food purchasing intention.

*H5*: Green self-identity positively influences sustainable food purchasing intention.

*H6*: Cognitive involvement mediates the relationship between green self-identity and sustainable food purchasing intention.

*H7*: Affective involvement mediates the relationship between green self-identity and sustainable food purchasing intention.

### 2.6 Generational differences

Generational differences are often invoked to explain variations in consumer behavior. However, they should not be treated as fixed psychological traits. Instead, they reflect shared socio-historical experiences that shape how individuals construct identity, interpret information, and engage with consumption practices.

In the context of sustainable consumption, these differences become particularly relevant. Prior research suggests that younger consumers vary in how sustainability is understood and enacted. Generation Z, for instance, is more likely to approach consumption as a form of identity expression, especially in digitally mediated environments where behaviors are visible and socially evaluated (Dabija et al., 2019; Becerra et al., 2023). In contrast, Generation Y tends to place relatively greater emphasis on information credibility and functional evaluation when making consumption decisions (Kumar et al., 2023).

Importantly, these differences do not imply that one generation is inherently more cognitive or more emotional than the other. Rather, they point to differences in how sustainability-related cues are processed and internalized. In socially visible contexts, social comparison processes (Festinger, 1954) can amplify identity expression, making affective engagement more salient. At the same time, signaling processes (Spence, 1973) continue to reduce uncertainty and support cognitive evaluation.

This suggests that cognitive and affective mechanisms are both activated in sustainable consumption, but their relative salience may vary across generational contexts. In other words, generational differences are better understood as boundary conditions that shape how identity is translated into behavior, rather than as deterministic explanations of behavior itself.

Accordingly, this study conceptualizes generational differences as moderating conditions within the identity-based dual-mechanism framework. Specifically, the strength of the relationship between green self-identity and sustainable food purchasing intention is expected to vary across generations. In addition, the effect of affective involvement on purchasing intention may differ depending on generational context. Accordingly, the following hypotheses are proposed:

*H8*: Generational differences moderate the relationship between green self-identity and sustainable food purchasing intention.

*H9*: Generational differences moderate the relationship between affective involvement and sustainable food purchasing intention.

## 3 Methodology

### 3.1 Participants and research design

This study adopts a cross-sectional survey design to examine how identity-based psychological mechanisms translate into sustainable food purchasing intention, with a particular focus on how cognitive evaluation and affective internalization jointly shape behavioral outcomes. The empirical context comprises Generation Y and Generation Z consumers in Taiwan, whose sustainability-related decisions reflect differences in identity formation and engagement with sustainability cues.

Data were collected through a convenience sampling strategy over a two-month period (January–February 2024). The online questionnaire was distributed via social media platforms and university mailing lists. Participation was voluntary and anonymous. Prior to the main survey, a pilot test with 30 participants was conducted to refine item clarity and contextual relevance. A total of 592 responses were collected. After removing incomplete or invalid questionnaires, 533 valid responses were retained, yielding a valid response rate of 90.03%.

To ensure ethical compliance, participation was restricted to adults aged 18 and above through a mandatory screening question. The study involved no sensitive topics and collected no personally identifiable information. According to Taiwan’s Human Subjects Research Act and relevant regulatory guidelines, such anonymous, minimal-risk survey research is exempt from formal Institutional Review Board (IRB) review. All participants received informed consent information prior to participation.

While this sampling approach facilitates efficient data collection, it may introduce self-selection bias, as individuals with stronger sustainability interest are more likely to participate. This limitation is acknowledged in interpreting the findings.

**Figure 1:**
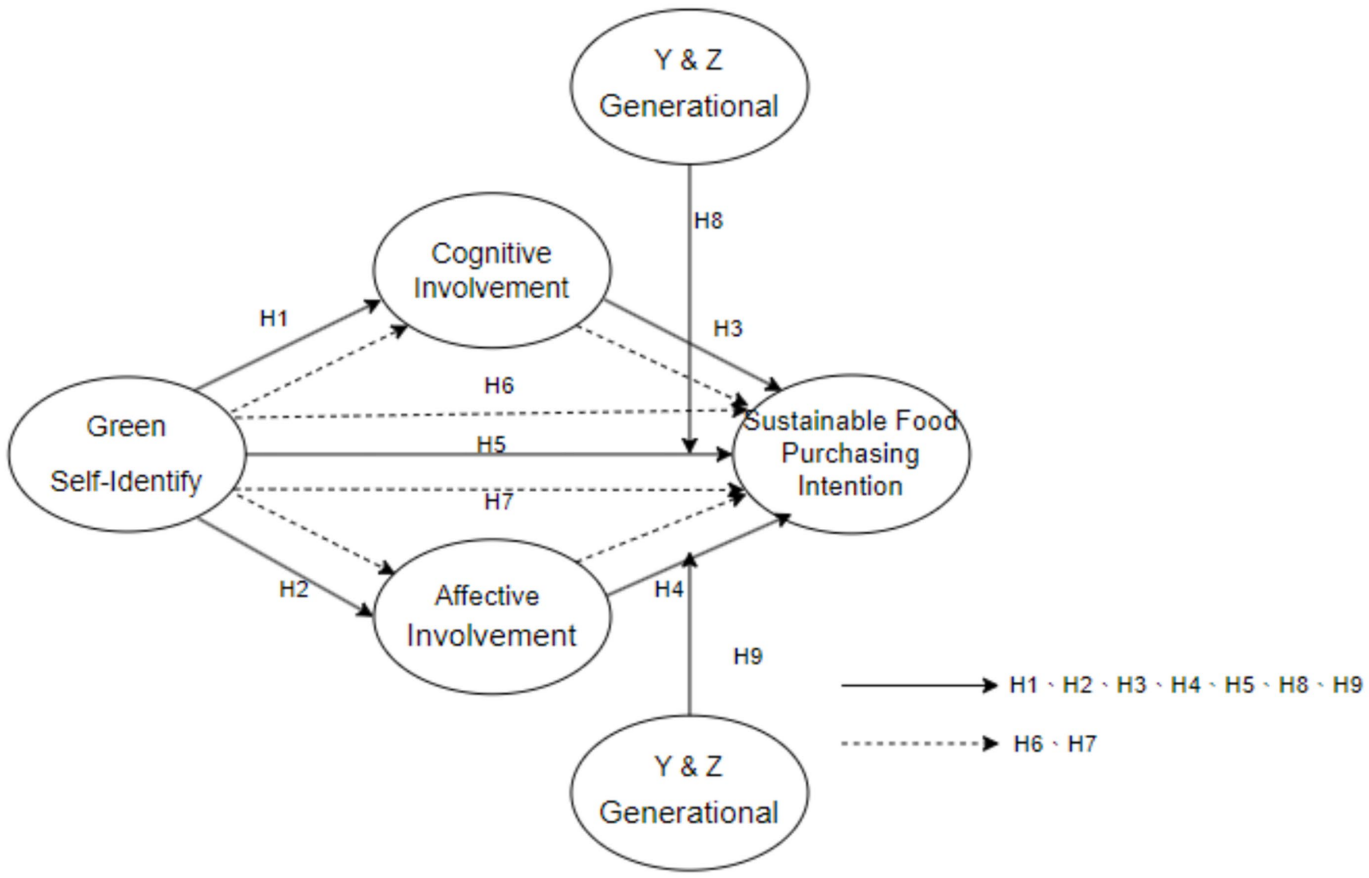
Research framework.

Based on the conceptual framework presented in Figure 1, this study examines the hypothesized relationships among green self-identity, cognitive involvement, affective involvement, and sustainable food purchasing intention, as well as the moderating role of generational differences.

Given that the conceptual model involves multiple latent constructs and seeks to capture both mediation and moderation processes, Structural Equation Modeling (SEM) was employed as the primary analytical approach. CFA was first conducted to establish measurement validity, followed by structural model estimation to test the hypothesized relationships. Mediation effects were examined using bootstrapping procedures, while moderation effects were assessed through multi-group SEM (MGSEM), enabling direct comparison of structural mechanisms across generational cohorts.

### 3.2 Measures and reliability analysis

All constructs were measured using multi-item Likert-type scales on a six-point scale ranging from 1 (“strongly disagree”) to 6 (“strongly agree”). All measurement items were adapted from validated scales in prior literature and modified to fit the context of sustainable food consumption.

Green self-identity (GSI) was measured using a five-item scale adapted from Griffin and O’Cass (2004). Cognitive involvement (CI) and affective involvement (AI) were measured using items adapted from Zaichkowsky’s (1994) Personal Involvement Inventory, capturing analytical evaluation and emotional engagement, respectively. Sustainable food purchasing intention (SFPI) was measured using a three-item scale developed by Hellier et al. (2003). Internal consistency was assessed using Cronbach’s alpha, with all constructs exceeding the recommended threshold of 0.70, indicating satisfactory reliability.

Item purification was conducted during CFA based on three criteria: (1) standardized factor loadings ≥ 0.70; (2) absence of substantial cross-loadings (> 0.35); and (3) Average Variance Extracted (AVE) ≥ 0.50.

Green self-identity (GSI) was initially measured using five items; two items were removed due to cross-loading, resulting in a final three-item construct. Cognitive involvement (CI) was measured using three items, all of which were retained. Affective involvement (AI) was initially measured using five items; two items were removed due to cross-loading, resulting in a final three-item construct. Sustainable food purchasing intention (SFPI) was measured using three items, all of which were retained. These final item counts correspond to those reported in Table 1.

**Table 1:** The results of convergent validity analysis.

Concept	Item	Standard factor loading	Composite reliability	Average variance extracted	Cronbach’s *α*	GSI	CI	AI	SFPI
GSI	GSI1	0.892	0.856	0.669	0.888	0.818*			
	GSI2	0.630							
	GSI4	0.903							
CI	CI1	0.868	0.869	0. 691	0.795	0.438**	0.831*		
	CI2	0.667							
	CI3	0.707							
AI	AI3	0.850	0.864	0.680	0.845	0.403**	0.539**	0.825*	
	AI4	0.849							
	AI5	0.665							
SFPI	SFPI 1	0.852	0.863	0.678	0.869	0.025	0.044	0.060	0.823*
	SFPI 2	0.855							
	SFPI 3	0.758							

*n* = 533. *Diagonal values represent the square root of the average variance extracted (√AVE). **Off-diagonal values represent correlations between constructs.

Following item purification, all constructs demonstrated satisfactory convergent validity, with standardized factor loadings exceeding 0.70, composite reliability (CR > 0.60), and Average Variance Extracted (AVE > 0.50) meeting recommended thresholds (Bagozzi and Yi, 1988). Discriminant validity was established using the Fornell–Larcker criterion (Fornell and Larcker, 1981).

To ensure the validity of generational comparisons, measurement invariance across Generation Y and Generation Z was tested using multi-group CFA (Vandenberg and Lance, 2000). Measurement invariance was assessed using configural, metric, and scalar models.

To address potential common method bias (CMB), three complementary procedures were employed: Harman’s single-factor test, a marker variable technique, and a CFA-based unmeasured latent factor approach (Lindell and Whitney, 2001; Podsakoff et al., 2003). These procedures were used to assess whether common method variance influenced the observed relationships.

## 4 Results

### 4.1 Sample profile

A total of 533 valid questionnaires were collected, yielding an effective response rate of 90.03%. Among the respondents, 45.2% belonged to Generation Y and 54.8% to Generation Z. Approximately 73.0% reported prior experience purchasing sustainable food products. Most respondents held a bachelor’s or master’s degree (90.2%), and 72% were unmarried. This sample composition provides an appropriate basis for examining identity-driven cognitive and affective mechanisms across generational cohorts.

### 4.2 Common method bias and measurement invariance

To assess the potential influence of common method bias (CMB), Harman’s single-factor test was first conducted using unrotated principal component analysis. The first factor accounted for 41.07% of the total variance, below the recommended threshold of 50%, suggesting that no single factor dominated the measurement structure.

To strengthen this assessment, two additional procedures were applied. First, the marker variable technique was employed using a theoretically unrelated variable (Lindell and Whitney, 2001). After controlling for shared variance, the magnitude and significance of the structural paths remained stable (maximum |Δβ| = 0.03). Second, a CFA-based unmeasured latent factor (ULF) approach was implemented (Podsakoff et al., 2003). The inclusion of the latent factor resulted in negligible changes in model fit (ΔCFI = 0.008; ΔRMSEA = 0.003), and substantive factor loadings remained consistent. Together, these results indicate that common method bias is unlikely to threaten the validity of the findings.

Prior to multi-group comparisons, measurement invariance across Generation Y and Generation Z was examined using multi-group CFA (MGCFA; Vandenberg and Lance, 2000). Three nested invariance models were evaluated sequentially. First, the configural model demonstrated acceptable fit (χ^2^/df = 2.21, CFI = 0.961, RMSEA = 0.048), supporting a consistent factor structure across groups. Second, the metric invariance model showed no significant deterioration in fit (ΔCFI = 0.008 < 0.010; Δχ^2^(12) = 18.43, *p* = 0.103), indicating equivalence of factor loadings. Third, the scalar invariance model also met recommended criteria (ΔCFI = 0.007 < 0.010; Δχ^2^(12) = 17.61, *p* = 0.128), confirming equivalence of item intercepts.

These results establish full measurement invariance, providing the necessary statistical foundation for the multi-group structural equation modeling reported in Section 4.5.

### 4.3 Measurement model assessment

#### 4.3.1 Convergent validity

Consistent with the procedures described in Section 3.2, confirmatory factor analysis (CFA) was conducted to evaluate the adequacy of the measurement model. Convergent validity was assessed using standardized factor loadings, composite reliability (CR), and average variance extracted (AVE). All retained items exhibited factor loadings above 0.60, CR values exceeded 0.60, and AVE values were greater than 0.50, indicating satisfactory convergent validity. Cronbach’s alpha values for all constructs exceeded the 0.70 threshold, confirming internal consistency. These results indicate that green self-identity, cognitive involvement, affective involvement, and sustainable food purchasing intention are empirically distinguishable and reliably measured psychological constructs.

#### 4.3.2 Discriminant validity

Discriminant validity was assessed using the Fornell–Larcker criterion. For each construct, the square root of the AVE exceeded its correlations with other constructs, indicating adequate discriminant validity (see Table 1). This finding supports the conceptual distinction between cognitive and affective involvement as separate psychological mechanisms.

It should be noted that Table 1 reports bivariate correlations among constructs, whereas the SEM results in Table 2 reflect structural relationships estimated while accounting for the full model.

**Table 2:** The results of SEM path analysis.

Hypothesis	Path		*β* (Standardized)	S.E.	C.R.	*p*-value
	Independent variable	Dependent variable				
H1	GSI	CI	0.317	0.038	11.831	***
H2	GSI	AI	0.398	0.042	9.755	***
H3	CI	SFPI	0.123	0.038	2.503	*
H4	AI	SFPI	0.237	0.047	4.380	***
H5	GSI	SFPI	0.505	0.039	13.1064	***

***p < 0.001, **p < 0.01, *p < 0.05.

#### 4.3.3 Structural equation modeling results

Structural equation modeling (SEM) was employed to test the proposed identity-based dual-mechanism framework. The structural model demonstrated acceptable fit (χ^2^/df = 2.21, CFI = 0.961, RMSEA = 0.048), indicating that the theoretical model adequately represents the data.

**Figure 2:**
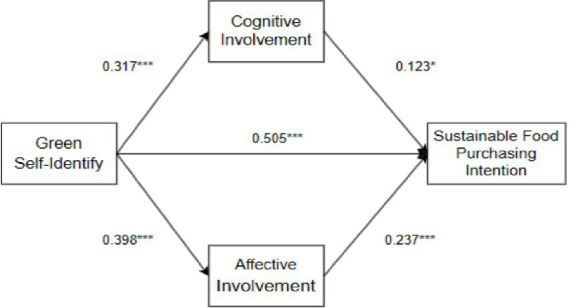
Structural equation model with standardized path coefficients. ***p < 0.001; *p < 0.05.

As presented in Table 2, all standardized path coefficients are positive and statistically significant (*p* < 0.05). Figure 2 provides a visual representation of the structural model using the same standardized estimates, illustrating the pattern of relationships reported in Table 2. All coefficients reported in both Table 2 and Figure 2 are standardized.

These findings provide empirical support for the proposed framework, demonstrating that identity influences behavior through both cognitive evaluation and affective engagement mechanisms.

### 4.4 Mediating effects of cognitive and affective mechanisms

The mediating roles of cognitive and affective involvement were examined using bootstrapping procedures with 5,000 resamples. The results indicate that cognitive involvement partially mediates the relationship between green self-identity and sustainable food purchasing intention (indirect effect = 0.150, 95% CI [0.081, 0.226]). Affective involvement also serves as a significant partial mediator (indirect effect = 0.087, 95% CI [0.045, 0.150]).

These findings indicate that identity operates through dual psychological pathways. Cognitive involvement provides an evaluative basis for decision-making, while affective involvement reflects emotional alignment with sustainability values. Together, these mechanisms explain how abstract identity is translated into concrete behavioral intention.

### 4.5 Moderating effects of generational differences

To examine the moderating role of generational differences, multi-group structural equation modeling (MGSEM) was conducted.

Following the establishment of measurement invariance (Section 4.2), structural path coefficients were compared across Generation Y and Generation Z. A baseline model with freely estimated parameters was compared with constrained models in which individual structural paths were set equal across groups. Differences in model fit were evaluated using Δχ^2^ and ΔCFI criteria.

The results indicate significant generational differences in several key structural relationships. First, the effect of green self-identity on sustainable food purchasing intention differs across groups, with a stronger effect observed for Generation Z (*β* = 0.612) than for Generation Y (*β* = 0.482), supported by a significant chi-square difference (Δχ^2^(1) = 5.83, *p* = 0.016).

Second, the effect of affective involvement on sustainable food purchasing intention is also stronger for Generation Z (*β* = 0.394) compared to Generation Y (*β* = 0.247), with a significant difference (Δχ^2^(1) = 9.21, *p* = 0.002).

In contrast, the effect of cognitive involvement on sustainable food purchasing intention is stronger for Generation Y (*β* = 0.341) than for Generation Z (*β* = 0.218), with the difference reaching statistical significance (Δχ^2^(1) = 4.47, *p* = 0.035).

These findings indicate that generational differences condition the relative strength of identity-based cognitive and affective mechanisms. While both generations rely on green self-identity as a foundational driver, Generation Z is more strongly associated with affective pathways, whereas Generation Y exhibits a comparatively stronger reliance on cognitive evaluation.

**Figure 3:**
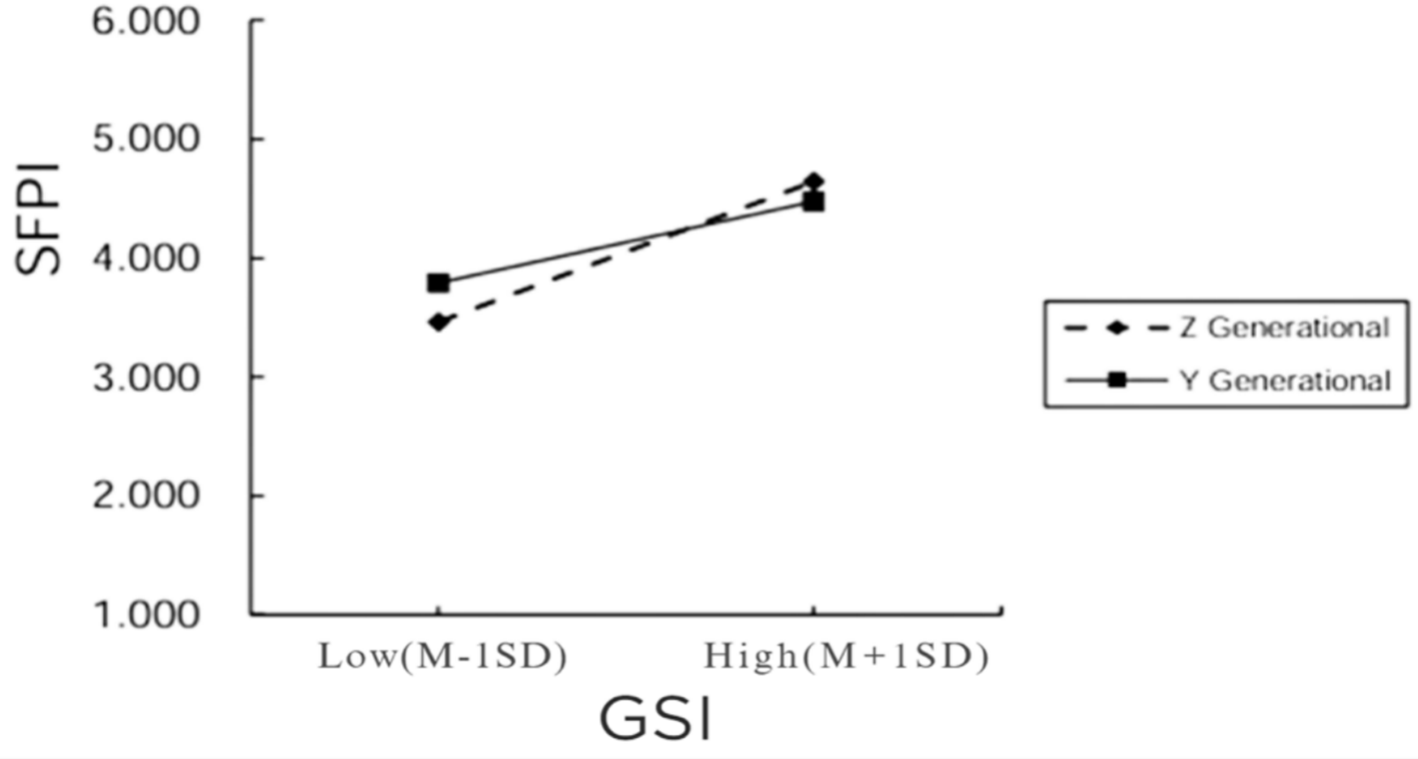
Group differences in the effect of green self-identity on sustainable food purchasing intention based on multi-group SEM. GSI, green self-identity; SFPI, sustainable food purchasing intention.

**Figure 4:**
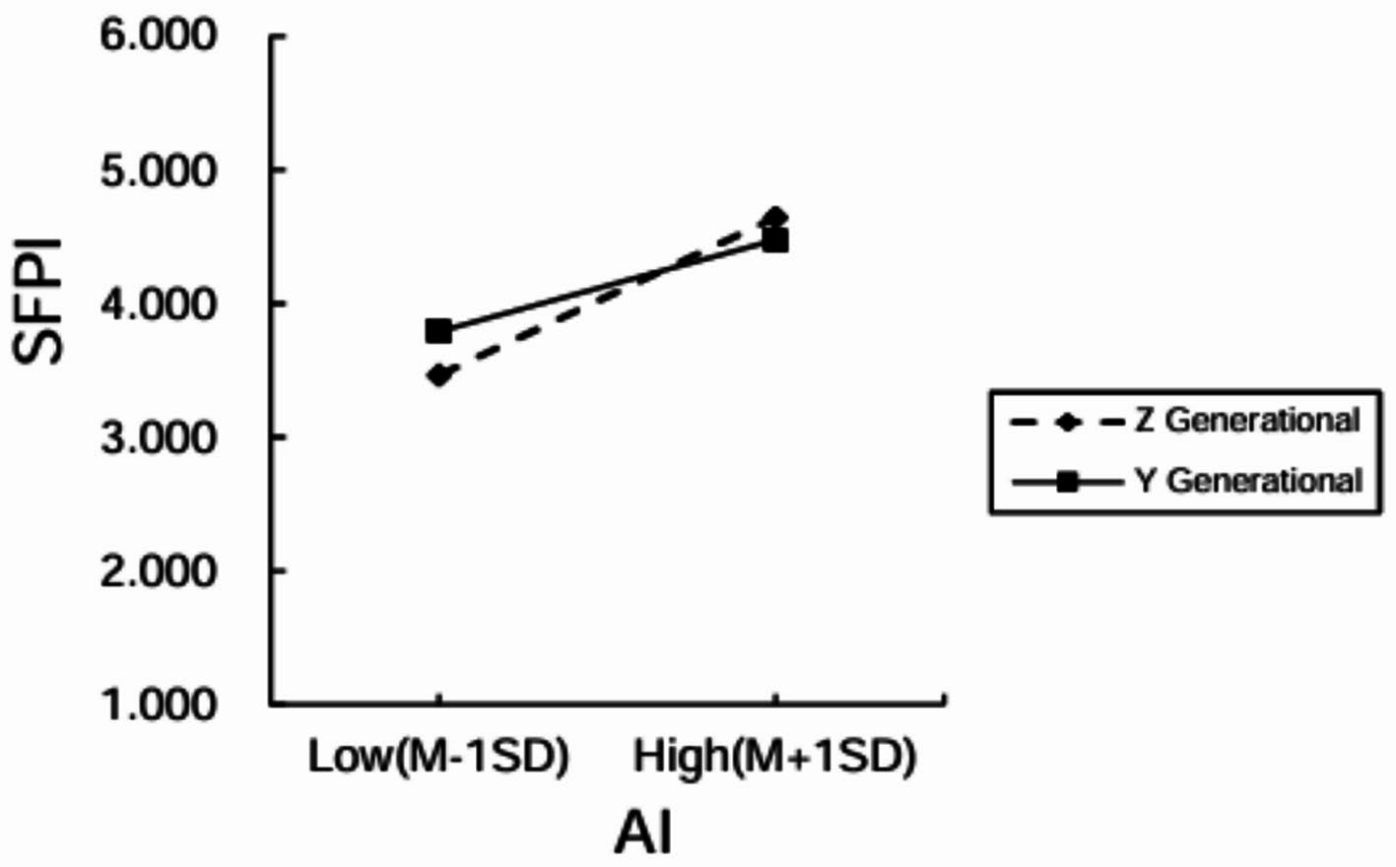
Group differences in the effect of affective involvement on sustainable food purchasing intention based on multi-group SEM. AI, Affective involvement; SFPI, Sustainable food purchasing intention.

Figures 3, 4 visually illustrate these group-specific effects. The plotted slopes are based on the estimated group-specific structural coefficients derived from MGSEM.

## 5 Discussion

This study advances the psychological understanding of sustainable food consumption by clarifying how green self-identity (GSI) is translated into purchasing intention through distinct yet interrelated cognitive and affective mechanisms. Rather than treating sustainable consumption as a purely attitudinal or informational outcome, the findings demonstrate that identity-consistent decision-making operates through both analytical evaluation and emotional resonance. This mechanism-based perspective helps explain the persistent intention–behavior gap, showing that pro-environmental values require both cognitive validation and emotional internalization to be translated into action.

By distinguishing between direct identity effects and indirect effects mediated through cognitive and affective involvement, the results provide empirical support for a dual-mechanism framework. Cognitive involvement reflects deliberate and effortful processing of sustainability-related information, such as evaluating eco-label credibility and environmental claims, whereas affective involvement captures emotional alignment and moral attachment to sustainability values. Importantly, these mechanisms operate in a complementary manner: cognitive evaluation provides a legitimacy basis for judgment, while affective involvement transforms such evaluation into emotionally meaningful commitment (Cancellieri et al., 2022). Together, they explain how eco-labels function not only as informational cues but also as psychologically meaningful signals that activate identity-consistent responses.

Importantly, the relative salience of these mechanisms varies across generational groups. The results suggest that, within the proposed model, Generation Y shows a relatively stronger reliance on cognitive involvement when forming sustainable food purchasing intentions, whereas Generation Z demonstrates a comparatively stronger association with affective involvement. This finding should be interpreted as a difference in pathway strength rather than as a fixed cognitive or emotional orientation. As the present study does not directly measure cognitive or affective traits, the interpretation is limited to structural relationships observed within the model. This distinction provides a more precise account of how “thinking” and “feeling” operate as differentiated routes through which identity influences sustainable behavior.

Cultural context further conditions these mechanisms. In Taiwan, where collectivist norms emphasize social harmony, moral obligation, and sensitivity to peer evaluation, green self-identity is more likely to be enacted through socially embedded consumption practices. Sustainable food choices therefore function not only as personal ethical expressions but also as relational behaviors shaped by social comparison and shared norms (Festinger, 1954). This context-sensitive interpretation underscores the importance of situating identity-based mechanisms within specific socio-cultural environments rather than assuming universal decision-making processes.

Taken together, the findings highlight the central role of identity-based psychological mechanisms—particularly green self-identity and affective involvement—in shaping sustainable food purchasing intention. While these mechanisms operate at the individual level, they also provide insight into how stable and socially reinforced patterns of sustainable consumption emerge.

### 5.1 Theoretical contribution 1—extension of signaling theory

This study extends Signaling Theory (Spence, 1973) by demonstrating that eco-labels operate as dual-function signals in sustainable consumption contexts. Specifically, eco-labels function as cognitive credibility signals that reduce information asymmetry and facilitate analytical evaluation, while simultaneously serving as symbolic cues that support identity expression and social positioning (Berger and Heath, 2007). By linking these dual functions to distinct psychological mechanisms, this study clarifies how signaling processes operate beyond information transmission and become embedded in identity-driven consumption.

### 5.2 Theoretical contribution 2—integration of signaling and social comparison theories

The findings demonstrate that Signaling Theory and Social Comparison Theory are not competing explanations but complementary frameworks operating through distinct yet interconnected pathways. Signaling Theory primarily explains the cognitive pathway (eco-label → evaluation → intention), whereas Social Comparison Theory explains the affective pathway (social visibility → identity resonance → affective involvement → intention) (Festinger, 1954). By integrating these perspectives within a unified model, this study provides a mechanism-based explanation of how external sustainability cues simultaneously activate analytical processing and emotional engagement.

### 5.3 Theoretical contribution 3—generational boundary conditions for involvement effects

The multi-group SEM results reveal that the relative strength of cognitive and affective pathways is contingent upon generational membership. This finding qualifies prior assumptions regarding the universal dominance of affective involvement in sustainable consumption (Arvola et al., 2008) by demonstrating that such effects are context-dependent. The identification of generational boundary conditions contributes to involvement theory by introducing a moderating perspective, suggesting that the effectiveness of cognitive versus affective mechanisms varies across socio-demographic contexts.

## 6 Conclusions and applications

### 6.1 Conclusion

By comparing Generation Y and Generation Z, this study demonstrates how identity-driven psychological mechanisms shape sustainable food purchasing intention in generationally distinct ways. Within the proposed model, Generation Z shows a relatively stronger association between affective involvement and sustainable food purchasing intention, whereas Generation Y shows a relatively stronger association between cognitive involvement and purchasing intention.

These findings suggest that sustainable consumption is not driven by a single motivational logic. Instead, identity-based decision-making unfolds through differentiated cognitive and affective mechanisms, with generational context influencing the relative salience of each pathway. Understanding these differences is important for explaining persistent intention–behavior gaps in sustainable food consumption.

### 6.2 Theoretical implications

This study contributes to theory by integrating green self-identity, involvement theory, signaling theory, and social comparison theory into a coherent psychological framework. By empirically demonstrating that cognitive and affective involvement operate as distinct yet interrelated mechanisms linking identity to behavior, the findings extend existing models of sustainable consumption beyond attitudinal explanations.

The generational contrast further advances theory by showing that sustainability cues may be processed through different psychological routes. For Generation Z, affective and identity-related pathways appear more salient, whereas for Generation Y, cognitive evaluation appears relatively more influential. This distinction enriches social comparison theory by illustrating how identity expression and emotional engagement may shape sustainable behavior in digitally mediated environments.

### 6.3 Managerial implications

From a managerial perspective, the findings suggest that sustainability communication strategies should be tailored to generationally differentiated psychological mechanisms. For Generation Z, emotionally resonant and identity-oriented messaging may be particularly effective when embedded in social and digital contexts. For Generation Y, transparent and information-rich communication emphasizing certification credibility and product attributes may be more persuasive.

Consistent and credible eco-labeling systems play a crucial role in converting identity-based motivation into stable purchasing intention. Firms can further strengthen this process by aligning organizational sustainability narratives with consumer identity concerns and by leveraging peer influence through social media engagement.

### 6.4 Policy implications

At the policy level, the results indicate that demand-side behavioral mechanisms can complement regulatory approaches to sustainable food systems. Policies that support standardized eco-labeling, transparent certification, and consumer education can enhance the effectiveness of identity-driven consumption, particularly among younger cohorts.

### 6.5 Limitations and future research

Several limitations warrant consideration. First, the cross-sectional design limits causal inference. Future research could employ longitudinal or experimental designs to capture the dynamic development of green self-identity and its psychological mechanisms. Second, the focus on Taiwanese consumers limits generalizability; cross-cultural studies would help clarify how cultural norms shape identity-based decision processes. Because the present study primarily focuses on theory-driven latent psychological mechanisms, demographic variables were not modeled as competing predictors in the structural model. Future research could further incorporate demographic and contextual variables to examine their additional explanatory value.

Future research should also examine how digital platforms influence green identity formation and the balance between cognitive and affective involvement over time. Integrating behavioral data or experimental measures would further strengthen understanding of how identity-driven mechanisms translate into actual purchasing behavior.

## Data Availability

The original contributions presented in the study are included in the article/supplementary material, further inquiries can be directed to the corresponding authors.

## References

[ref1] Abubakari A. Majeed M. Amoako G. K. Ofori K. S. Ampong G. O. A. (2025). Going green, paying more: the relationship between trust in green labeling, green perceived value and price premiums. Int. J. Qual. Serv. Sci. 17, 381–404. doi: 10.1108/ijqss-09-2023-0132

[ref2] Andrade E. B. Vieites Y. (2025). Obstacles and opportunities for sustainable consumption: a comprehensive conceptual model, literature review, and research agenda. J. Consum. Psychol. 35, 637–662. doi: 10.1002/jcpy.70003

[ref3] Arvola A. Vassallo M. Dean M. Lampila P. Saba A. Lähteenmäki L. . (2008). Predicting intentions to purchase organic food: the role of affective and moral attitudes in the theory of planned behaviour. Appetite 50, 443–454. doi: 10.1016/j.appet.2007.09.010, 18036702

[ref4] Bagozzi R. P. Yi Y. (1988). On the evaluation of structural equation models. J. Acad. Mark. Sci. 16, 74–94. doi: 10.1007/bf02723327

[ref5] Becerra E. P. Carrete L. Arroyo P. (2023). A study of the antecedents and effects of green self-identity on green behavioral intentions of young adults. J. Bus. Res. 155:113380. doi: 10.1016/j.jbusres.2022.113380

[ref6] Berger J. Heath C. (2007). Where consumers diverge from others: identity signaling and product domains. J. Consum. Res. 34, 121–134. doi: 10.1086/519142

[ref7] Cancellieri U. G. Petruccelli I. Cicero L. Milani A. Bonaiuto F. Bonaiuto M. (2022). Reputation and emotion: how the mind drives our food preferences and choices. Food Qual. Prefer. 101:104637. doi: 10.1016/j.foodqual.2022.104637

[ref8] Chen Y. S. Chang C. H. (2012). Enhance green purchase intentions: the roles of green perceived value, green perceived risk, and green trust. Manag. Decis. 50, 502–520. doi: 10.1108/00251741211216250

[ref9] Dabija D. C. Bejan B. M. Dinu V. (2019). How sustainability oriented is generation Z in retail? A literature review. Transform. Bus. Econ. 18. Available online at: https://www.transformations.knf.vu.lt/47/article/hows

[ref10] Festinger L. (1954). A theory of social comparison processes. Hum. Relat. 7, 117–140. doi: 10.1177/001872675400700202

[ref11] Fornell C. Larcker D. F. (1981). Evaluating structural equation models with unobservable variables and measurement error. J. Mark. Res. 18, 39–50. doi: 10.2307/3151312

[ref12] Griffin D. O’Cass A. (2004). Social marketing: who really gets the message? J. Nonprofit Publ. Sect. Market. 12, 129–147. doi: 10.1300/J054v12n02_07

[ref13] Grunert K. G. Hieke S. Wills J. (2014). Sustainability labels on food products: consumer motivation, understanding and use. Food Policy 44, 177–189. doi: 10.1016/j.foodpol.2013.12.001

[ref14] Hamby A. Jones N. (2022). The effect of affect: an appraisal theory perspective on emotional engagement in narrative persuasion. J. Advert. 51, 116–131. doi: 10.1080/00913367.2021.1981498

[ref16] Hellier P. K. Geursen G. M. Carr R. A. Rickard J. A. (2003). Customer repurchase intention: a general structural equation model. Eur. J. Mark. 37, 1762–1800. doi: 10.1108/03090560310495456

[ref17] Hou C. Sarigöllü E. (2022). Is bigger better? How the scale effect influences green purchase intention: the case of washing machine. J. Retail. Consum. Serv. 65:102894. doi: 10.1016/j.jretconser.2021.102894

[ref18] Hu M. L. M. Horng J. S. Teng C. C. Chiou W. B. Yen C. D. (2014). Fueling green dining intention: the self-completion theory perspective. Asia Pacific J. Tour. Res. 19, 793–808. doi: 10.1080/10941665.2013.806941

[ref19] Jang Y. J. Kim E. (2024). How self-identity and social identity grow environmentally sustainable restaurants’ brand communities via social rewards. J. Hospital. Tour. Res. 48, 516–532. doi: 10.1177/10963480221140019

[ref20] Kovač I. Dunković D. Kovač B. (2025). Greenwashing and consumer skepticism toward eco-labels in Croatia: challenges and policy directions. Br. Food J. [Ahead of print]. doi: 10.1108/BFJ-02-2025-0122

[ref21] Kumar R. Kumar K. Singh R. Sá J. C. Carvalho S. Santos G. (2023). Modeling environmentally conscious purchase behavior: examining the role of ethical obligation and green self-identity. Sustainability 15:6426. doi: 10.3390/su15086426

[ref22] Langaro D. Loureiro S. M. C. Brântuas M. (2025). Are eco-labels able to go beyond signaling environmental benefits? Using eco-labels to communicate the economic value of sustainability in food products. J. Strateg. Mark. 34, 92–112. doi: 10.1080/0965254X.2025.2584043

[ref23] Lau M. M. Ng P. M. L. Chan E. A. H. Cheung C. T. Y. (2023). Examining purchase intention for luxury fashion: integrating theory of reasoned action, with affect-behavior-cognition (ABC) model, identity and social identity theories. Young Consum. 24, 114–131.

[ref24] Lindell M. K. Whitney D. J. (2001). Accounting for common method variance in cross-sectional research designs. J. Appl. Psychol. 86, 114–121. doi: 10.1037/0021-9010.86.1.114, 11302223

[ref9002] Lin S. T. Niu H. J. (2018). Green consumption: Environmental knowledge, environmental consciousness, social norms, and purchasing behavior. Business Strategy and the Environment, 27, 1679–1688.

[ref25] Mahasuweerachai P. Suttikun C. (2022). The effect of green self-identity on perceived image, warm glow and willingness to purchase: a new generation’s perspective towards eco-friendly restaurants. Sustainability 14:10539. doi: 10.3390/su141710539

[ref26] McAdams D. P. (1995). What do we know when we know a person? J. Pers. 63, 365–396. doi: 10.1111/j.1467-6494.1995.tb00500.x

[ref9001] Mehta A. Sharma M. Gupta N. (2024). Determinants responsible for sustainable consumption behavior among youths. SocioEconomic Challenges, 8, 240–252.

[ref27] Nguyen K. H. Tran M. D. Ho V. T. (2026). Mapping the nexus between sustainable consumption and corporate social responsibility: intellectual structure, evolution and research agenda. Corp. Soc. Responsib. Environ. Manag. 1–20. doi: 10.1002/csr.70476

[ref28] Niu H. J. Chen M. J. (2022). Exploring the co-creation value of residents to tourists from the perspective of place attachment and economic benefits. Front. Psychol. 13:877365. doi: 10.3389/fpsyg.2022.877365, 35664140 PMC9159473

[ref29] Niu H. J. Hsieh K. Y. Hsieh F. Y. Li M. H. Yu C. C. Lin C. T. (2025a). Beyond attitude: the moderating role of sport and leisure involvement in green food consumption and sustainable agriculture. J. East Eur. Manag. Stud. 30:40221. doi: 10.31083/JEEMS40221

[ref30] Niu H. J. Wu E. T. Yen C. Y. Chen M. J. Yu C. C. (2025b). From visitors to vitality: how relational populations support regional revitalization in aging urban and rural areas. Sustain. Futures 9:100669. doi: 10.1016/j.sftr.2025.100669

[ref31] Petty R. E. Cacioppo J. T. Schumann D. (1983). Central and peripheral routes to advertising effectiveness: the moderating role of involvement. J. Consum. Res. 10, 135–146. doi: 10.1086/208954

[ref32] Podsakoff P. M. MacKenzie S. B. Lee J. Y. Podsakoff N. P. (2003). Common method biases in behavioral research: a critical review of the literature and recommended remedies. J. Appl. Psychol. 88, 879–903. doi: 10.1037/0021-9010.88.5.879, 14516251

[ref33] Rex E. Baumann H. (2007). Beyond ecolabels: what green marketing can learn from conventional marketing. J. Clean. Prod. 15, 567–576. doi: 10.1016/j.jclepro.2006.05.013

[ref35] Skinner E. A. Belmont M. J. (1993). Motivation in the classroom: reciprocal effects of teacher behavior and student engagement across the school year. J. Educ. Psychol. 85:571. doi: 10.1037/0022-0663.85.4.571

[ref36] Spence M. (1973). Job market signaling. Q. J. Econ. 87, 355–374. doi: 10.2307/1882010

[ref39] Vandenberg R. J. Lance C. E. (2000). A review and synthesis of the measurement invariance literature. Organ. Res. Methods 3, 4–70.

[ref38] Van der Werff E. Steg L. Keizer K. (2013). The value of environmental self-identity: the relationship between biospheric values, environmental self-identity and environmental preferences, intentions and behaviour. J. Environ. Psychol. 34, 55–63. doi: 10.1016/j.jenvp.2012.12.006

[ref40] Whitmarsh L. O’Neill S. (2010). Green identity, green living? The role of pro-environmental self-identity in determining consistency across diverse pro-environmental behaviours. J. Environ. Psychol. 30, 305–314. doi: 10.1016/j.jenvp.2010.01.003, 38826717

[ref41] Zaichkowsky J. L. (1985). Measuring the involvement construct. J. Consum. Res. 12, 341–352.

[ref42] Zaichkowsky J. L. (1994). The personal involvement inventory: reduction, revision, and application to advertising. J. Advert. 23, 59–70. doi: 10.1080/00913367.1943.10673459

[ref43] Zheng C. Ling S. Cho D. (2023). How social identity affects green food purchase intention: the serial mediation effect of green perceived value and psychological distance. Behav. Sci. 13:664. doi: 10.3390/bs13080664, 37622804 PMC10451480

